# Brownian Motion at Lipid Membranes: A Comparison of Hydrodynamic Models Describing and Experiments Quantifying Diffusion within Lipid Bilayers

**DOI:** 10.3390/biom8020030

**Published:** 2018-05-22

**Authors:** Stephan Block

**Affiliations:** Department of Chemistry and Biochemistry, Freie Universität Berlin, D-14195 Berlin, Germany; stephan.block@fu-berlin.de; Tel.: +49-30-838-60071

**Keywords:** lipid bilayer, membrane proteins, membrane hydrodynamics, diffusion, Saffman-Delbrück, Evans-Sackmann, Hughes-Pailthorpe-White

## Abstract

The capability of lipid bilayers to exhibit fluid-phase behavior is a fascinating property, which enables, for example, membrane-associated components, such as lipids (domains) and transmembrane proteins, to diffuse within the membrane. These diffusion processes are of paramount importance for cells, as they are for example involved in cell signaling processes or the recycling of membrane components, but also for recently developed analytical approaches, which use differences in the mobility for certain analytical purposes, such as in-membrane purification of membrane proteins or the analysis of multivalent interactions. Here, models describing the Brownian motion of membrane inclusions (lipids, peptides, proteins, and complexes thereof) in model bilayers (giant unilamellar vesicles, black lipid membranes, supported lipid bilayers) are summarized and model predictions are compared with the available experimental data, thereby allowing for evaluating the validity of the introduced models. It will be shown that models describing the diffusion in freestanding (Saffman-Delbrück and Hughes-Pailthorpe-White model) and supported bilayers (the Evans-Sackmann model) are well supported by experiments, though only few experimental studies have been published so far for the latter case, calling for additional tests to reach the same level of experimental confirmation that is currently available for the case of freestanding bilayers.

## 1. Introduction

**Figure 1:**
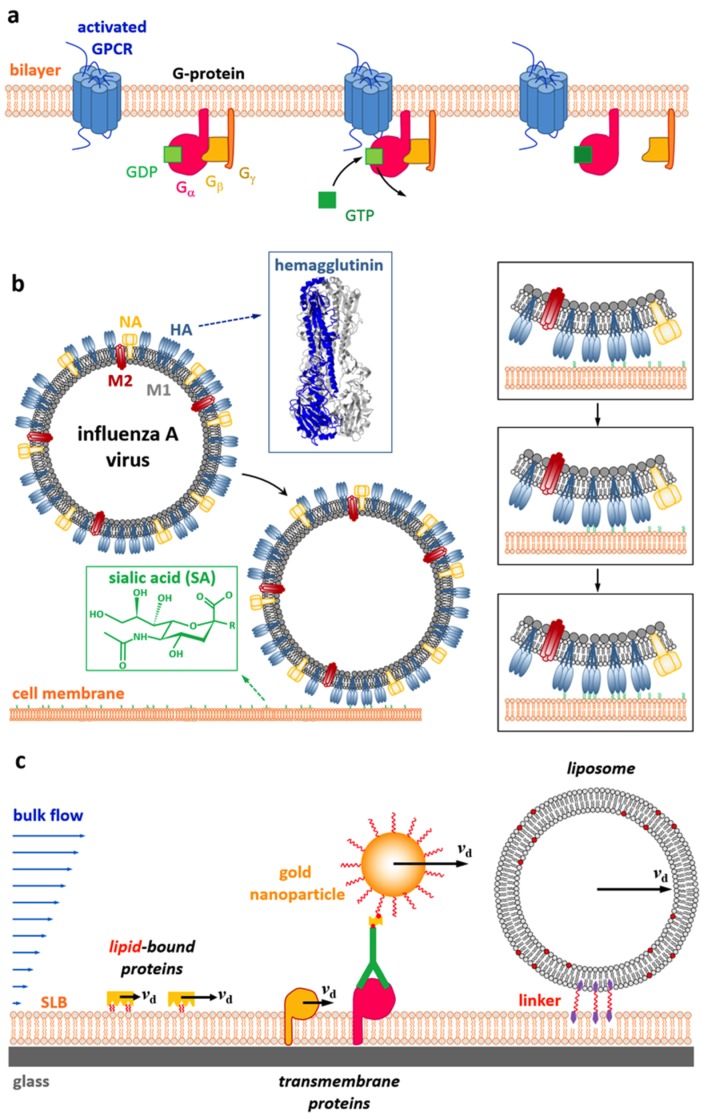
Diffusion of bilayer-bound membrane components is a phenomenon ubiquitously observed in nature. (**a**) Many cell signaling processes rely on the formation of membrane complexes, an example of which is given here by the activation of a trimeric G-protein by a G-protein coupled receptor (GPCR). The GPCR itself may be activated by the binding of a ligand or the isomerization of an incorporated co-factor. Once activated, the GPCR promotes the exchange of a guanosine diphosphate (GDP) with a guanosine triphosphate (GTP) in an interacting G-protein, which in turn causes the G-protein to dissociate into its α- and βγ-subunits and thus transducing the signal into the cell’s interior using enzymatic activities of the released α-subunit. The interaction of the GPCR with the G-protein as well as dissociation and re-association of the G-protein subunits obviously requires them to be mobile within the bilayer. (**b**) Diffusion also plays an important role for many multivalent interactions, such as virus–receptor interactions occurring during virus entry. Receptor-mediated virus diffusion is, for example, expected to be one way to increase the overall virus–membrane interaction by subsequently increasing the number of receptors engaged by the virus (see insets), a process that is promoted by diffusion of the virus and/or the membrane-bound receptors (HA: hemagglutinin; PDB code 1RD8). (**c**) Differences in the mobility or size of membrane-associated objects (e.g., proteins, liposomes) can also be used for analytical purposes in the presence of a hydrodynamic shear force (e.g., created by a microfluidic environment). This concept has been used in the past to separate lipid-bound proteins based on a difference in friction experienced at the supported lipid bilayer (SLB), to separate transmembrane proteins in near-native membrane bilayers by specifically tagging proteins of interest using antibody-linked nanoparticles, and to characterize bilayer-linked structures, such as liposomes, based on an analysis of the shear force-induced drift velocity $v_{d}$ (the length of which has been indicated in panel (**c**) only for illustrative purposes).

Bilayers that are formed by self-assembly of lipid molecules represent one of the major building blocks found in nature [1]. They act often as nm-thin hydrophobic barriers that allow for cells to compartmentalize into distinct regions and host proteins that facilitate transport of material (e.g., nutrients, wastes, and metabolites) along membranes or between different cell compartments [2]. Interestingly, lipid bilayers usually provide a fluidic functionality, in the sense that proteins that are embedded in or carbohydrates attached to cell membranes can diffuse laterally in the bilayers [3], with diffusion coefficients typically ranging between 10^−3^ and 2 μm^2^/s (depending on the membrane composition, the presence of interactions to additional cellular structures, such as the cell’s cytoskeleton, the size of the diffusing object, etc., [4]). In fact, many biological processes are known to heavily rely on this lateral diffusion, that is, on the mobility of membrane components [1]. For example, the cross-linking of receptors is a feature that is often observed in cell signaling processes and it requires the presence of receptors that are able to move within the membrane (Figure 1a) [5,6].

Furthermore, viruses, which are known to bind to cell membranes via the engagement of multiple membrane-bound receptors, often show diffusion after the initial formation of the first bonds (Figure 1b), which is often interpreted as a search for additional receptors (allowing to increase the interaction to the membrane), and thus for optimal regions for the entry process [7,8,9,10].

The importance of diffusion in such processes stimulated intense research, aiming to improve the theoretical understanding of the interplay between, e.g., receptor diffusion and biological function, and to establish new experimental methods allowing for analyzing biological processes based on measuring the mobility of the involved membrane-associated compounds. Of particular interest is the question, how lipids and proteins diffuse in general within bilayers, and therefore how the mobility of a membrane inclusion (e.g., created by cross-linking of membrane proteins) depends on its size, the knowledge of which would for example allow for measuring the oligomerization of membrane receptors based on a measurement of their mobility. Direct application of the developed theories to cell membranes, however, turned out to be very challenging, indicating that cell membranes possess much larger complexity than is covered by most theoretical models [3,11,12]. For example, continuum theories predict a rather small decrease of the receptor mobility upon receptor dimerization [13], which is in stark contrast to most of the experimental observations that were done on the membranes of living cells [14]. These and related observations stimulated the creation of more complex models of the cell membrane (see e.g., References [3,11,12] for recent reviews), which, on the other hand, are very challenging to analyze using theoretical approaches.

Nevertheless, several model systems, such as giant unilamellar vesicles (GUVs) [15], supported lipid bilayers (SLBs) [1], and free-standing membranes (so-called black lipid membranes, BLMs) [16,17] have been introduced in the past, which can be considered as minimalistic mimics of cellular membranes as they provide a fluid, lipid bilayer-based environment allowing to host membrane-associated proteins while maintaining their native functionality. In fact, as these model systems are far less complex than cell membranes, they finally allowed for probing the validity of models describing the diffusion of lipids and proteins within bilayers; the results of these efforts will be summarized in this review. While this, in principle, allows for some of the initial questions to be addressed in well controlled model systems, it was realized, in addition, that the fluidic functionality of bilayers can also be used for other analytical purposes, such as the (electrophoretically or hydrodynamically driven) isolation of membrane-associated compounds or transmembrane proteins [18,19,20,21], the characterization of bilayer-linked objects [20,22,23,24], or the quantification of multivalent interactions [10]. The validity of these new analytical approaches also requires a profound knowledge about the principle of diffusion processes that take place within bilayers and hence, this field benefited strongly from the theoretical considerations mostly done in the 1980s and 1990s.

This review aims to summarize the different theories developed to describe diffusion within lipid bilayers and to provide a comparison between the theoretical predictions and recent experimental demonstrations using the aforementioned model systems. Lipids, peptides, proteins, and complexes thereof will be approximated by cylindrical inclusions having inclusion radii >0.5 nm and performing Brownian motion (i.e., normal diffusion). It will be shown that models describing diffusion of such inclusions in freestanding bilayers (i.e., the Saffman-Delbrück and the Hughes-Pailthorpe-White model) have been mostly confirmed experimentally with respect to variations in the inclusion radius, while the case of bilayers being supported by solid substrates has been less well addressed experimentally.

## 2. Models of Membrane Hydrodynamics

**Figure 2:**
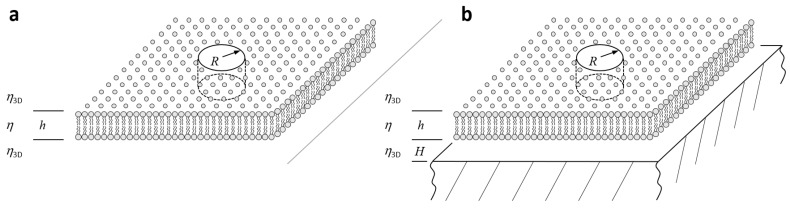
Schematic illustration of a cylindrical membrane inclusion (radius R) diffusing within a bilayer (viscosity η, thickness h). The bilayer membrane is either embedded in an incompressible bulk fluid with viscosity $\eta_{3D}$ (**a**—freestanding bilayer, observed for giant unilamellar vesicles, giant unilamellar vesicle (GUVs), or black lipid membranes (BLMs)) or supported by a solid substrate (**b**—supported lipid bilayer (SLB); $H<\ell_{m}=\eta h/2\eta_{3D}$). The distance between SLB and support, H, can range between 1 and ~100 nm, depending on the nature and composition of the lubricating layer introduced between the membrane (bare versus polymer-supported versus droplet interface bilayers) and the substrate. In principle, (**a**) follows from (**b**) in the limit of $H>>\ell_{m}$.

This section gives a summary of continuum theories describing the Brownian motion (i.e., normal diffusion) of lipids, peptides, proteins, and complexes thereof within lipid bilayers. These objects are approximated by cylindrical inclusions (with radius R) that are embedded within a sheet of thickness h and viscosity η, representing the lipid bilayer (Figure 2a). Hence, the entire molecular structure of the bilayer is ignored and is approximated by a two-dimensional (2D) continuum. The general goal of these theoretical considerations was to arrive at an expression for the translational or rotational friction coefficient, $f_{t}$ and $f_{r}$, respectively, which can be related to the translational ($D_{t}$) or rotational ($D_{r}$) diffusion coefficient using the Einstein relations:

(1) $$D_{t}=k_{B}T/f_{t}\mathrm{and}D_{r}=k_{B}T/f_{r},$$

with $k_{B}$ denoting Boltzmann’s constant and T the temperature [13]. Furthermore, both friction coefficients can also be related to the corresponding mobilities:

(2) $$\mu_{t}=1/f_{t}\mathrm{and}\mu_{r}=1/f_{r},$$

which are defined as the drift or angular velocity generated upon action of a steady unit force or torque, respectively [13].

First attempts to directly solve the linearized Navier-Stokes equation for the 2D motion of such a viscous, incompressible fluid around a resting cylindrical inclusion failed, as it was not possible to simultaneously satisfy all boundary conditions of the problem, leading to the so-called Stokes paradox. Saffman and Delbrück realized that this paradox is avoided by embedding the 2D continuum into an incompressible three-dimensional (3D) medium having a lower yet finite viscosity $\eta_{3D}$ (Figure 2a) [13].

Subsequent analysis showed that this procedure allows for a momentum exchange to take place between the bilayer and the surrounding medium, thereby yielding a solution to the linearized Navier-Stokes equation satisfying all of the boundary conditions simultaneously. For the geometry that is depicted in Figure 2a, that is, for a freestanding bilayer being embedded into a bulk medium, Saffman and Delbrück calculated this solution using single perturbation technique to the leading order [13], yielding:

(3) $$f_{t}=4\pi \eta h{\left\{\ln\frac{2}{\varepsilon }-\gamma \right\}}^{-1}\mathrm{and}f_{r}=4\pi \eta hR^{2},$$

with γ=0.5772 denoting Euler’s constant and introducing the dimensionless parameter ε defined by:

(4) $$\varepsilon =\frac{R}{h}\frac{2\eta_{3D}}{\eta }.$$

Further analysis showed that Equation (3) yields a good approximation of the exact solution for ε≤0.1 and that the ratio R/ε corresponds the characteristic length scale, $\ell_{m}=R/\varepsilon =\eta h/2\eta_{3D}$, at which the bilayer starts to exchange momentum with the surrounding bulk medium [25,26]. Hence, in contrast to bulk hydrodynamics, which is a scale-free theory at low Reynolds numbers, the hydrodynamic coupling between the membrane and the bulk fluid introduces even at low Reynolds numbers a characteristic length scale $\ell_{m}$ that formally corresponds to the membrane thickness times the viscosity contrast arising between the membrane and the bulk fluid. As will be shown in the Section 3, the typical values for the viscosity of fluid phase lipid bilayers are on the order of η=0.1 Pa⋅s=1 P, which lead (together with a water viscosity of $\eta_{3D}=1\;\mathrm{mPa}\cdot s$ and a typical bilayer thickness of h=5 nm) to $\ell_{m}\propto 250\;\mathrm{nm}$; a value that can increase even further when studying bilayers of larger viscosity.

In order to derive a solution for larger values of ε, Hughes et al. [25] calculated further terms to the asymptotic expansion of Equation (3), leading to:

(5) $$f_{t}=4\pi \eta h{\left\{\ln\frac{2}{\varepsilon }-\gamma +\frac{4}{\varepsilon }-\frac{\varepsilon^{2}}{2}\ln\frac{2}{\varepsilon }\right\}}^{-1}\mathrm{and}f_{r}=4\pi \eta hR^{2}{\left\{1+\frac{8}{3\pi }\varepsilon \right\}}^{-1},$$

which is a good approximation for ε≤1. Furthermore, they also calculated the asymptotic solutions for large ε,

(6) $$f_{t}\propto \eta h\varepsilon \mathrm{and}f_{r}\propto \eta h\varepsilon R^{2},$$

and provided a numerical solution of the problem for all ε [25]. Equation (6) predicts the diffusion coefficients of very large inclusions ($R>>\ell_{m}$) to become independent on bilayer viscosity η and to decay as $D_{t}\propto 1/R$ and $D_{r}\propto 1/R^{3}$, respectively. Petrov et al. [27,28] finally used the numerical solution that was derived by Hughes and co-workers to arrive at an empirical equation that allows for $f_{t}$ and $f_{r}$ to be calculated with high accuracy for ε ranging between 10^−3^ and 10^3^:

(7) $$f_{t}=4\pi \eta h{\left\{\ln\frac{2}{\varepsilon }-\gamma +\frac{4\varepsilon }{\pi }-\frac{\varepsilon^{2}}{2}\ln\frac{2}{\varepsilon }\right\}}^{-1}\left\{1-\frac{\varepsilon^{3}}{\pi }\ln\frac{2}{\varepsilon }+\beta \left(\varepsilon ,b_{t1},b_{t2},c_{t1},c_{t2}\right)\right\}\mathrm{and}f_{r}=\pi \frac{{\left(\eta h\right)}^{3}}{\eta_{3D}^{2}}\left\{\varepsilon^{2}+\frac{4\varepsilon^{3}}{3\pi }+\beta \left(\varepsilon ,b_{r1},b_{r2},c_{r1},c_{r2}\right)\right\},$$

using the bridging function

$$\beta \left(\varepsilon ,p,q,v,w\right)=v\varepsilon^{p}/\left(1+w\varepsilon^{q}\right),$$

and $b_{t1}=2.74819$, $b_{t2}=0.51465$, $c_{t1}=0.73761$, and $c_{t2}=0.52119$ for $f_{t}$ and $b_{r1}=2.91587$, $b_{r2}=0.68319$, $c_{r1}=0.31943$, and $c_{r2}=0.60737$ for $f_{r}$.

**Figure 3:**
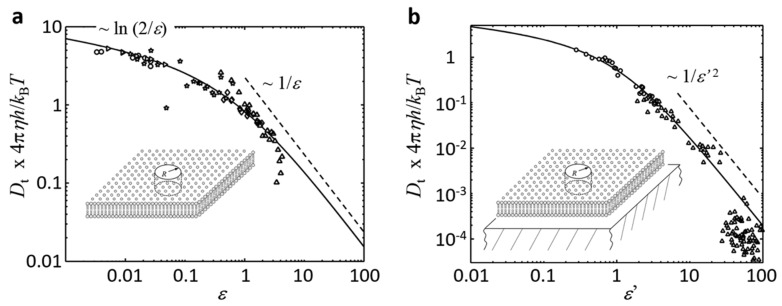
Dependence of reduced transversal diffusion coefficient $D_{t}\cdot 4\pi \eta h/k_{B}T$ from the reduced inclusion radius ε and ε′, respectively, for freestanding (**a**—$H>>\ell_{m}$; Hughes­Pailthorpe-White (HPW) model) or supported bilayers (**b**—$H<\ell_{m}$; Evans and Sackmann (ES) model). The solid lines give the solutions of the HPW (**a**) or ES model (**b**), respectively, while the data points originate from the publications summarized in Table 1 after rescaling using the published value of ηh (please refer to Table 1 for further information on the included data points). Although the published data can be well described by the HPW and ES model, respectively, it should be noted (as further discussed in Section 3.3) that up to now, only few studies are available that test the predictions of the ES model and that in these studies the experimental geometry is often more complex than is indicated by the inset. Parameter: R, inclusion radius.

As already mentioned in the beginning of this section, all of these theoretical considerations are only valid for bilayers that are freestanding within a bulk medium, that is, for bilayers that have surface separations H being much larger than $\ell_{m}$: $H>>h\cdot \eta /2\eta_{3D}$ (Figure 2). This condition is fulfilled for GUVs and BLMs, but not for SLBs, which will be discussed separately below. Figure 3a shows the reduced translational diffusion coefficient $D_{t}\cdot 4\pi \eta h/k_{B}T$ versus the reduced inclusion radius ε for ε ranging between 10^−3^ and 10^3^, showing the weak increase ~lnε for ε < 0.1 and the asymptotic scaling ~ε for ε > 10. This behaviour provides an alternative interpretation of the length scale $\ell_{m}$, in the sense that membrane inclusions appear as point-like structures to the surrounding flow for inclusion radii R being much smaller than $\ell_{m}$, causing a logarithmically weak increase in $f_{t}$ with increasing R. Since most of the membrane proteins are much smaller than $\ell_{m}$, the oligomerization of membrane proteins is therefore expected to cause only a weak increase in $f_{t}$, and thus a weak decrease in $D_{t}$.

Evans and Sackmann realized that this behaviour would strongly change if a solid support is closely located to the bilayer ($H<\ell_{m}$, Figure 2b), which is usually the case for lipid mono- and bi-layers being adsorbed to solid surfaces [26]. By considering a weak frictional coupling between bilayer and solid substrate, which is described by the friction coefficient $b_{s}$, they introduce another dimensionless parameter:

(8) $$\varepsilon \prime =R{\left\{\frac{b_{s}}{\eta h}\right\}}^{1/2},$$

and finally arrive at

(9) $$f_{t}=4\pi \eta h\left\{\frac{\varepsilon \prime^{2}}{4}\left(1+\frac{b_{p}}{b_{s}}\right)+\frac{\varepsilon \prime \cdot K_{1}(\varepsilon \prime )}{K_{0}(\varepsilon \prime )}\right\}\mathrm{and}f_{r}=4\pi \eta hR^{2}\left\{1+\frac{\varepsilon \prime }{2}\frac{K_{0}(\varepsilon \prime )}{K_{1}(\varepsilon \prime )}+\frac{b_{p}}{8b_{s}}\varepsilon \prime^{2}\right\},$$

using the modified Bessel function of second kind $K_{0}$ and $K_{1}$ and the coefficient of friction between the inclusion and the solid substrate $b_{p}$ [26]. For thin layers ($H<<\ell_{m}$), the friction coefficient $b_{s}$ can be approximated by $b_{s}=\eta_{3D}/H$, allowing for expressing ε′ in terms of ε:

(10) $$\varepsilon \prime =R{\left\{\frac{\eta_{3D}}{\eta hH}\right\}}^{1/2}={\left\{\frac{\varepsilon \;R}{2H}\right\}}^{1/2}.$$

Figure 3b shows the reduced translational diffusion coefficient $D_{t}\cdot 4\pi \eta h/k_{B}T$ of the Evans-Sackmann model versus the reduced inclusion radius ε′ for ε′ ranging between 10^−2^ and 10 and $b_{p}=b_{s}$, showing a weak increase for ε′ < 0.1 and the asymptotic scaling $\sim \varepsilon \prime^{2}$ for ε′ > 1. 

Inserting typical values (η=0.1 Pa⋅s,$\eta_{3D}=1\;\mathrm{mPa}\cdot s$, h=5 nm, and H=1 nm) indicates that the Evans-Sackmann model predicts a strong decrease in mobility ($\sim 1/R^{2}$) for membrane inclusions, with radii exceeding ~20 nm; a size that is much smaller than the length scale $\ell_{m}$. Hence, the coupling to the substrate located at a distance H introduces a second characteristic length scale $\ell_{H}$, which can be defined, based on the structure of Equation (10), according to $\ell_{H}={\left(2H\ell_{m}\right)}^{1/2}$ and is typically much smaller than $\ell_{m}$ for supported bilayers ($H<<\ell_{m}$) [40].

In addition to what was presented here, there have been extensions of the initial models, calculating, for example, the mobility of a viscous or anisotropic inclusion within a viscous membrane [41,42,43], the effect of crowding on the mobility of membrane inclusions [44], and the effect of (static or dynamic) membrane fluctuations on the motion of membrane inclusions [45,46,47,48,49].

## 3. Experimental Observations

The models that were introduced in the previous section provide several predictions that can be experimentally tested, allowing the applicability of the models to be evaluated. This section will summarize the experimental results (see Table 1), thereby allowing for comparing theoretical predictions and experimental observations. The model introduced by Saffman and Delbrück (SD) (Equation (3)) and extended by Hughes, Pailthorpe, and White (HPW) (Equation (5)) is, from a theoretical point of view, only valid for freestanding bilayers ($H>>\ell_{m}$); a condition that is experimentally accessible by studying Brownian motion in GUVs or BLMs. In contrast, the model derived by Evans and Sackmann (ES) (Equation (9)) requires a notable frictional coupling to a nearby substrate ($H<<\ell_{m}$), a condition that is fulfilled for example by SLBs, but also by droplet interface bilayers (DIB) as long as the substrate-bilayer distance H remains significantly smaller than $\ell_{m}$.

### 3.1. Freestanding Bilayers in the Limit of Small ε ($H>>\ell_{m}$, ε<0.1): Saffman and Delbrück

This limit of the SD model was experimentally addressed in the past by measuring the translational and rotation diffusion coefficients of proteins and cross-linked peptides of known size R in GUVs and BLMs. Equation (3) predicts in this case $D_{t}$ to scale as $\sim \ln\left(2\ell_{m}/R\right)$, that is, to show a weak logarithmic decrease with increasing R. First attempts indeed showed this weak decrease, but suffered from a low dynamic range of experimentally accessible ε values [29,50,51]. This problem was finally solved by Ramadurai et al. [30] and Weiss et al. [31], who reconstituted a variety of membrane proteins into the membranes of GUVs or BLMs, and quantified $D_{t}$ with high accuracy using fluorescence correlation spectroscopy (FCS). In these experiments, the inclusion radius R was estimated based on the molecular structure of the employed proteins, which was available from crystallographic measurements, allowing for fitting Equation (3) to the data points. Both works observed an excellent agreement between the SD model and the data, yielding reasonable values for the membrane viscosity η (see Table 1), and therefore confirming the applicability of the SD model for freestanding bilayers having inclusions being much smaller than $\ell_{m}$.

Nevertheless, it should be mentioned that the applicability of the SD model has been challenged in the past by experimental and theoretical studies, mainly discussing the influence of a hydrophobic (height) mismatch between the inclusion and the membrane, or the presence of membrane fluctuations on the observed diffusion coefficients. In this context, the work of Gambin et al. [38] is often referred to, who observe a 1/R scaling of $D_{t}$ instead of the $\sim \ln\left(2\ell_{m}/R\right)$ scaling predicted by SD. However, as pointed out by Ramadurai et al. [30], this discrepancy may arise from the presence of surface effects, caused by the interface that is employed by Gambin et al. [38] to measure diffusion in the GUVs using evanescent excitation [52]. Such a coupling to a nearby interface can well explain the much stronger scaling observed for $D_{t}$ [26]. Furthermore, numerical studies on the effect of a hydrophobic mismatch indeed showed a minor change in the effective membrane viscosity, but no fundamental changes in the general scaling behaviour of $D_{t}$ for small inclusions [53], making it unlikely that a hydrophobic mismatch alone explains the observed scaling.

### 3.2. Freestanding Bilayers in the Limit of Large ε ($H>>\ell_{m}$,ε>1): Hughes, Pailthorpe, and White

This limit of the HPW model was experimentally addressed in the past by measuring the translational and rotation diffusion coefficients of lipid domains that were formed in GUVs having binary or ternary lipid compositions. As the domains are well resolvable using optical microscopy, single particle tracking (SPT) can be used to determine the diffusion coefficients, while the domain size can be extracted from the microscopic images. Depending on composition and the phase of the bilayer surrounding the µm-sized lipid domains, $\ell_{m}$ ranged between 0.5 and 10 µm in these experiments (see Table 1), allowing for probing the HPW model in the limits of intermediate and large ε. For large ε (>10), Equation (6) predicts $D_{t}$ and $D_{r}$ to scale as 1/R and $1/R^{3}$, respectively, and to become independent of the membrane viscosity η. For intermediate ε the equation provided by Petrov and Schwille can be used to probe the test of the HPW model [27,28].

One of the first tests was conducted by Cicuta et al. [33], who studied the motion of lipid domains in GUVs consisting of different mixtures of 1,2-dioleoyl-sn-glycero-3-phosphocholine (DOPC), 1,2-dipalmitoyl-sn-glycero-3-phosphocholine (DPPC), and cholesterol. The motion of *L*_o_ phase DPPC domains in *L_α_* phase DOPC bilayers is expected to follow the large ε limit and the measured $D_{t}$ values indeed showed the 1/R scaling. In the opposite case, that is, when studying the motion of *L_α_* DOPC domains in *L*_o_ DPPC bilayers, the authors were able to change the viscosity η of the DPPC bilayers over a wide range by control of the measurement temperature, allowing for transitions between the intermediate and large ε limit to be observed in a single experiments. In this case, the authors were able to extract η values for DPPC bilayers that are up to three orders of magnitude larger than those that were observed for fluid phase bilayers (Table 1), being in agreement with other studies on the diffusion in gel-phase bilayers [4]. It should be noted, however, that in Cicuta et al. [33] and in a related study conducted by Stanich et al. [34], the authors did not fit the HPW model, which would be required when operating at intermediate ε values, but used a modified version of the SD model, which was derived using a different boundary condition and adds a +1/2 term to the bracket in Equation (3) [13]. Fitting instead the HPW model to the data leads to minor modifications of the extracted parameters, and it improves the description of the experimental data [27].

The validity of the HPW model is also demonstrated by Petrov et al. [28], who quantify $D_{t}$ and $D_{r}$ of diamond-shaped DPPC domains diffusing in fluid-phase 1,2-diphytanoyl-sn­glycero-3-phosphocholine (DPhPC) bilayers. They probed the case of intermediate ε values and observed excellent agreement between the HPW model, calculated using their own numerical solution to the problem, Equation (7), and the data derived for $D_{t}$, $D_{r}$, and the ratio $D_{t}/D_{r}$. The extracted viscosity of the DPhPC bilayer is larger than observed for DOPC and POPC (Table 1), which is in reasonable agreement with complementary studies of the diffusion in DOPC and DPhPC bilayers [54]. This and related studies therefore confirm the applicability of the HPW model for freestanding bilayers having inclusions of a size being comparable or larger than $\ell_{m}$.

### 3.3. Lipid Bilayers Close to a Support ($H<<\ell_{m}$): Evans and Sackmann

The ES model received, in comparison to the SD or HPW model, less attention in the past, and hence, only few studies have been reported that probe the predictions done by Equation (9). The expression for the rotational (diffusion or friction) coefficient has not been tested so far. A direct application of the concepts of the previous sections turned out to be very challenging, as transmembrane proteins, for example, tend to be immobile if incorporated in SLBs, which is often attributed to strong steric effects arising between their endo-/ectodomains and the supporting surface [1]. Nevertheless, as this problem was recently solved, for example, by inserting polymer cushions between the bilayer and substrate [55,56,57], measurements that are similar to those that were conducted by Ramadurai et al. [30] and Weiss et al. [31] could be possible now even when using SLBs.

Due to these restrictions, tests of the ES model often relied on crosslinking of lipids, peptides, or truncated proteins, which was done at the upper leaflet of the bilayer. Kaizuka et al. [36] studied, for example, the diffusion of µm-size junctions that were formed between a supported and a floating lipid bilayer. They observed two different types of junctions, corresponding to bilayer–bilayer separations of approximately 3 and 50 nm (determined using Förster resonance energy transfer (FRET) and interferometric approaches), and quantified their diffusion and size using fluorescence microscopy. The data can be well described with Equation (9), and shows the expected $\sim 1/R^{2}$ scaling of $D_{t}$ for ε′ >> 1, though it should be noted that the complicated bilayer geometry makes it challenging to decide if effects beyond the ES model (e.g., due to bilayer bending during movement of the junctions) play a notable role.

Zhang et al. [58] study the interactions of polymers with lipids and the effect of these interactions on the lipid diffusion within a SLB based on 1,2-dilauroyl-sn-glycero-3-phosphocholine (DLPC). Using FCS, two distinct mobility populations are observed, which are attributed to lipids either being bound to the added polymer ($D_{t}$ < 2 µm^2^/s) or freely diffusing within the SLB ($D_{t}$ = 2.6 µm^2^/s). Interestingly, the slowly diffusing population shows a clear decrease of $D_{t}$, with increasing degree of polymerization, N, of the added polymer, following a ~1/N scaling. This behaviour can, in principle, be understood using the ES model, if one assumes that the added polymers bind to lipids within an area of the SLB, the radius of which is given by the polymers’ radius of gyration, $R_{g}$. As $R_{g}$ scales roughly with $\sim N^{0.6}$ and $D_{t}\sim R^{-2}$ for large ε′ [59] the observed scaling of $D_{t}\sim N^{-1}$ is in agreement with the ES model. Fitting the ES model using estimates for $R_{g}$ of the respective polymers yields values of $b_{s}$ ~ 10^7^ Pa s/m and η ~ 0.1 nPa m s, which are one order of magnitude larger ($b_{s}$) or smaller (η) than expected, thereby showing the limitations of this analysis.

Nevertheless, an excellent agreement between the ES model and mobility data is provided by de Wit et al. [37], who studied the motion of sphingomyelin (SM) nanodomains in DOPC droplet interface bilayers (DIB) that were formed above a 100 nm thin hydrogel. Using the scattering approach, iSCAT, the authors are able to track the motion of SM nanodomains in a label-free fashion with nm-accuracy and to determine $D_{t}$ for nanodomains radii ranging between ~100 to 1000 nm. The data is well fitted by the ES model, yielding reasonable values for membrane viscosity and coefficient of friction. Furthermore, the data covers ε' values ranging between ~0.2 up to ~10, clearly showing the $D_{t}\sim R^{-2}$ scaling expected for large ε′, and therefore confirming the much larger size-induced decrease in diffusivity in comparison to the SD and HPW model.

Finally, it should be mentioned that recently studies have been reported, which seem to indicate that the $D_{t}\sim R^{-2}$ scaling is already observed when crosslinking single lipids or transmembrane proteins that are embedded in SLBs [10,60,61]. These studies show a $D_{t}\sim 1/n$ scaling of the translational diffusion coefficient with the number of cross-linked objects, n. This scaling is compatible with the $\sim R^{-2}$ scaling, basically indicating a decrease of $D_{t}$ with one that is divided by the cumulated area of the inclusions, which increases linearly with the number of cross-linked objects. Nevertheless, this interpretation requires values for the coefficients of friction $b_{s}$ and $b_{p}$ that are at least two orders of magnitude larger than theoretically expected. As the distance between the cross-linked objects was on the scale of few nm or more in these experiments, it is more likely that the objects can be regarded as cross-linked yet hydrodynamically independently diffusing objects, so that the so-called free-draining model appropriately describes the relationship between $D_{t}$ and n [60]. This interpretation was recently supported by a numerical study [62].

## 4. Conclusions

In conclusion, very good agreement has been achieved between the predictions of the Saffman-Delbrück and the Hughes-Pailthorpe-White model, and the observed diffusion coefficients of proteins ($D_{t}$) and lipid domains ($D_{t}$ and $D_{r}$) moving within freestanding bilayers (i.e., in GUVs and BLMs), in dependence of the radius of these membrane inclusions. Up to date, less experimental data is available for the technically very important case of supported bilayers (e.g., SLBs). For this case, reported studies show a good agreement ($D_{t}$) with the Evans-Sackmann model, but additional tests are necessary to reach the same level of experimental confirmation that is currently available for the freestanding bilayers.

## Figures and Tables

**Table 1:** Overview of experiments reporting agreement with the models of Saffman-Delbrück (SD), Hughes-Pailthorpe-White (HPW), and Evans-Sackmann model (ES), or reporting deviations in the expected scaling of the Saffman-Delbrück model (!SD). The column ηh indicates the product of bilayer thickness and viscosity as reported in the corresponding reference (in which it was extracted by fitting the respective hydrodynamic model to the experimental data).

Model	System	Bilayer	T/°C	Approach	*ηh*/(nNs/m)	Remarks	Ref.
SD	Peptides	DMPC; GUV	35	FRAP; *D*_t_	0.525	Low dynamic range in ε	[29]
SD	Proteins	DOPC + DOPG; GUV	n.s.	FCS; *D*_t_	0.304	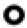 in Figure 3a	[30]
SD	Proteins	POPC + POPE; BLM	22	2fFCS; *D*_t_	0.15	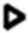 in Figure 3a	[31]
SD, HPW	Beads	DOPC; BLM	24	SPT; *D*_t_, *D*_r_	15.3 ± 3.4 (SD), 15.9 ± 2.3 (HPW)	Relatively high values extracted for *ηh*	[32]
SD	DOPC domains, 0.5–10 µm	DOPC + DPPC + Chol; GUV	16–30	SPT; *D*_t_	10–500	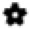 in Figure 3a	[33]
SD	DPPC domains, 1–10 µm	DPhPC + DPPC + Chol; GUV	26.2	SPT; *D*_t_	3.3 ± 1.1	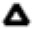 in Figure 3a	[34]
HPW	DPPC domains, 0.7–2 µm	DPhPC + DPPC (1:1); GUV	23.5	SPT; *D*_t_, *D*_r_	2.1–2.3	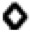 in Figure 3a	[28]
HPW	DPPC domains, 0.5–10 µm	DOPC + DPPC + Chol (2:2:1); GUV	16–30	SPT; *D*_t_	n.d.		[33]
ES	Crosslinked C10 chains	DMPC; multi-SLB	27–38	FRAP; *D*_t_	0.13–0.08	Low dynamic range in ε′	[35]
ES	Bilayer junctions	DMPC + DOTAP + PC (88:10:2); SLB	n.s.	SPT; *D*_t_	0.4	Complex bilayer geometry, 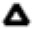 in Figure 3b	[36]
ES	SM nanodomains	DOPC + SM (1:1); DIB	n.s.	SPT; *D*_t_	0.87	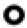 in Figure 3b	[37]
!SD	Peptides, proteins	SOPC, C12E5; GUV	20	FRAP; *D*_t_	n.s.	1/R instead ln(1/R) scaling, evanescent excitation	[38]
!SD	Proteins	POPC; GUV	25	2fFCS; *D*_t_	n.s.	1/R instead ln(1/R) scaling	[39]

^1^ Abbreviations: black lipid membrane (BLM), cholesterol (Chol), 1,2-dimyristoyl-sn-glycero-3-phosphocholine (DMPC), 1,2-dioleoyl-sn-glycero-3-phosphocholine (DOPC), 1,2-dioleoyl-sn-glycero-3-phosphoglycerol (DOPG), 1,2-dioleoyl-3-trimethylammonium-propane (DOTAP), 1,2-diphytanoyl-sn-glycero-3-phosphocholine (DPhPC), 1,2-dipalmitoyl-sn-glycero-3-phosphocholine (DPPC), fluorescence correlation spectroscopy (FCS), 2-focus FCS (2fFCS), fluorescence recovery after photobleaching (FRAP), giant unilamellar vesicle (GUV), not determined (n.d.), not specified (n.s.), phosphocholine (PC), 1-palmitoyl-2-oleoyl-SN-glycero-3-phosphocholine (POPC), 1-palmitoyl-2-oleoyl-sn-glycero-3-phosphoethanolamine (POPE), rotational diffusion coefficient (*D*_r_), supported lipid bilayer (SLB), sphingomyelin (SM), 1-stearoyl-2-oleoyl-sn-glycero-3-phosphocholine (SOPC), single particle tracking (SPT), translational diffusion coefficient (*D*_t_).

## References

[1] Tanaka M., Sackmann E. (2005). Polymer-supported membranes as models of the cell surface. Nature.

[2] Stein W.D., Lieb W.R. (1986). Transport and Diffusion across Cell Membranes.

[3] Kusumi A., Nakada C., Ritchie K., Murase K., Suzuki K., Murakoshi H., Kasai R.S., Kondo J., Fujiwara T. (2005). Paradigm shift of the plasma membrane concept from the two-dimensional continuum fluid to the partitioned fluid: High-speed single-molecule tracking of membrane molecules. Annu. Rev. Biophys. Biomol. Struct..

[4] Tocanne J.F., Dupoucezanne L., Lopez A. (1994). Lateral diffusion of lipids in model and natural membranes. Prog. Lipid Res..

[5] Irannejad R., von Zastrow M. (2014). GPCR signaling along the endocytic pathway. Curr. Opin. Cell Biol..

[6] Metzger H., Kinet J.P. (1988). How antibodies work—Focus on Fc-receptors. FASEB J..

[7] Mercer J., Schelhaas M., Helenius A. (2010). Virus entry by endocytosis. Annu. Rev. Biochem..

[8] Barrow E., Nicola A.V., Liu J. (2013). Multiscale perspectives of virus entry via endocytosis. Virol. J..

[9] Mammen M., Choi S.K., Whitesides G.M. (1998). Polyvalent interactions in biological systems: Implications for design and use of multivalent ligands and inhibitors. Angew. Chem. Int. Ed..

[10] Block S., Zhdanov V.P., Höök F. (2016). Quantification of multivalent interactions by tracking single biological nanoparticle mobility on a lipid membrane. Nano Lett..

[11] Bernardino de la Serna J., Schutz G.J., Eggeling C., Cebecauer M. (2016). There is no simple model of the plasma membrane organization. Front. Cell Dev. Biol..

[12] Metzler R., Jeon J.H., Cherstvy A.G. (2016). Non-brownian diffusion in lipid membranes: Experiments and simulations. Biochim. Biophys. Acta.

[13] Saffman P.G., Delbruck M. (1975). Brownian-motion in biological-membranes. Proc. Natl. Acad. Sci. USA.

[14] Iino R., Koyama I., Kusumi A. (2001). Single molecule imaging of green fluorescent proteins in living cells: E-cadherin forms oligomers on the free cell surface. Biophys. J..

[15] Dimova R., Aranda S., Bezlyepkina N., Nikolov V., Riske K.A., Lipowsky R. (2006). A practical guide to giant vesicles. Probing the membrane nanoregime via optical microscopy. J. Phys. Condens. Matter.

[16] Tien H.T., Diana A.L. (1968). Bimolecular lipid membranes—A review and a summary of some recent studies. Chem. Phys. Lipids.

[17] Winterhalter M. (2000). Black lipid membranes. Curr. Opin. Colloid Interface Sci..

[18] Yoshina-Ishii C., Boxer S.G. (2006). Controlling two-dimensional tethered vesicle motion using an electric field: Interplay of electrophoresis and electro-osmosis. Langmuir.

[19] Jönsson P., Gunnarsson A., Höök F. (2011). Accumulation and separation of membrane-bound proteins using hydrodynamic forces. Anal. Chem..

[20] Lundgren A., Fast B.J., Block S., Agnarsson B., Reimhult E., Gunnarsson A., Hook F. (2018). Affinity purification and single-molecule analysis of integral membrane proteins from crude cell-membrane preparations. Nano Lett..

[21] Gunnarsson A., Nystrom L.S., Burazerovic S., Gunnarsson J., Snijder A., Geschwindner S., Hook F. (2016). Affinity capturing and surface enrichment of a membrane protein embedded in a continuous supported lipid bilayer. Chemistryopen.

[22] Bally M., Gunnarsson A., Svensson L., Larson G., Zhdanov V.P., Höök F. (2011). Interaction of single virus-like particles with vesicles containing glycosphingolipids. Phys. Rev. Lett..

[23] Block S., Fast B.J., Lundgren A., Zhdanov V.P., Höök F. (2016). Two-dimensional flow nanometry of biological nanoparticles for accurate determination of their size and emission intensity. Nat. Commun..

[24] Tabaei S.R., Gillissen J.J.J., Block S., Hook F., Cho N.J. (2016). Hydrodynamic propulsion of liposomes electrostatically attracted to a lipid membrane reveals size-dependent conformational changes. ACS Nano.

[25] Hughes B.D., Pailthorpe B.A., White L.R. (1981). The translational and rotational drag on a cylinder moving in a membrane. J. Fluid Mech..

[26] Evans E., Sackmann E. (1988). Translational and rotational drag coefficients for a disk moving in a liquid membrane-associated with a rigid substrate. J. Fluid Mech..

[27] Petrov E.P., Schwille P. (2008). Translational diffusion in lipid membranes beyond the Saffman-Delbruck approximation. Biophys. J..

[28] Petrov E.P., Petrosyan R., Schwille P. (2012). Translational and rotational diffusion of micrometer-sized solid domains in lipid membranes. Soft Matter.

[29] Lee C.C., Petersen N.O. (2003). The lateral diffusion of selectively aggregated peptides in giant unilamellar vesicles. Biophys. J..

[30] Ramadurai S., Holt A., Krasnikov V., van den Bogaart G., Killian J.A., Poolman B. (2009). Lateral diffusion of membrane proteins. J. Am. Chem. Soc..

[31] Weiss K., Neef A., Van Q., Kramer S., Gregor I., Enderlein J. (2013). Quantifying the diffusion of membrane proteins and peptides in black lipid membranes with 2-focus fluorescence correlation spectroscopy. Biophys. J..

[32] Hormel T.T., Kurihara S.Q., Brennan M.K., Wozniak M.C., Parthasarathy R. (2014). Measuring lipid membrane viscosity using rotational and translational probe diffusion. Phys. Rev. Lett..

[33] Cicuta P., Keller S.L., Veatch S.L. (2007). Diffusion of liquid domains in lipid bilayer membranes. J. Phys. Chem. B.

[34] Stanich C.A., Honerkamp-Smith A.R., Putzel G.G., Warth C.S., Lamprecht A.K., Mandal P., Mann E., Hua T.A.D., Keller S.L. (2013). Coarsening dynamics of domains in lipid membranes. Biophys. J..

[35] Liu C.H., Paprica A., Petersen N.O. (1997). Effects of size of macrocyclic polyamides on their rate of diffusion in model membranes. Biophys. J..

[36] Kaizuka Y., Groves J.T. (2004). Structure and dynamics of supported intermembrane junctions. Biophys. J..

[37] De Wit G., Danial J.S.H., Kukura P., Wallace M.I. (2015). Dynamic label-free imaging of lipid nanodomains. Proc. Natl. Acad. Sci. USA.

[38] Gambin Y., Lopez-Esparza R., Reffay M., Sierecki E., Gov N.S., Genest M., Hodges R.S., Urbach W. (2006). Lateral mobility of proteins in liquid membranes revisited. Proc. Natl. Acad. Sci. USA.

[39] Kriegsmann J., Gregor I., von der Hocht I., Klare J.P., Engelhard M., Enderlein J., Fitter J. (2009). Translational diffusion and interaction of a photoreceptor and its cognate transducer observed in giant unilamellar vesicles by using dual-focus FCS. ChemBioChem.

[40] Stone H.A., Ajdari A. (1998). Hydrodynamics of particles embedded in a flat surfactant layer overlying a subphase of finite depth. J. Fluid Mech..

[41] Ramachandran S., Komura S., Imai M., Seki K. (2010). Drag coefficient of a liquid domain in a two-dimensional membrane. Eur. Phys. J. E.

[42] Levine A.J., Liverpool T.B., MacKintosh F.C. (2004). Mobility of extended bodies in viscous films and membranes. Phys. Rev. E.

[43] Levine A.J., Liverpool T.B., MacKintosh F.C. (2004). Dynamics of rigid and flexible extended bodies in viscous films and membranes. Phys. Rev. Lett..

[44] Oppenheimer N., Diamant H. (2009). Correlated diffusion of membrane proteins and their effect on membrane viscosity. Biophys. J..

[45] Reister E., Seifert U. (2005). Lateral diffusion of a protein on a fluctuating membrane. Europhys. Lett..

[46] Gov N.S. (2006). Diffusion in curved fluid membranes. Phys. Rev. E.

[47] Henle M.L., Levine A.J. (2010). Hydrodynamics in curved membranes: The effect of geometry on particulate mobility. Phys. Rev. E.

[48] Naji A., Brown F.L.H. (2007). Diffusion on ruffled membrane surfaces. J. Chem. Phys..

[49] Naji A., Levine A.J., Pincus P.A. (2007). Corrections to the Saffman-Delbruck mobility for membrane bound proteins. Biophys. J..

[50] Criado M., Vaz W.L.C., Barrantes F.J., Jovin T.M. (1982). Translational diffusion of acetylcholine-receptor (monomeric and dimeric forms) of torpedo-marmorata reconstituted into phospholipid-bilayers studied by fluorescence recovery after photobleaching. Biochemistry.

[51] Vaz W.L.C., Criado M. (1985). A comparison of the translational diffusion of a monomer and an oligomer of the acetylcholine-receptor protein reconstituted into soybean lipid bilayers. Biochim. Biophys. Acta.

[52] Gambin Y., Reffay M., Sierecki E., Homble F., Hodges R.S., Gov N.S., Taulier N., Urbach W. (2010). Variation of the lateral mobility of transmembrane peptides with hydrophobic mismatch. J. Phys. Chem. B.

[53] Guigas G., Weiss M. (2008). Influence of hydrophobic mismatching on membrane protein diffusion. Biophys. J..

[54] Shenoy S., Moldovan R., Fitzpatrick J., Vanderah D.J., Deserno M., Losche M. (2010). In-plane homogeneity and lipid dynamics in tethered bilayer lipid membranes (tBLMs). Soft Matter.

[55] Sackmann E., Tanaka M. (2000). Supported membranes on soft polymer cushions: Fabrication, characterization and applications. Trends Biotechnol..

[56] Sinner E.K., Knoll W. (2001). Functional tethered membranes. Curr. Opin. Chem. Biol..

[57] Pace H., Nystrom L.S., Gunnarsson A., Eck E., Monson C., Geschwindner S., Snijder A., Hook F. (2015). Preserved transmembrane protein mobility in polymer-supported lipid bilayers derived from cell membranes. Anal. Chem..

[58] Zhang L.F., Granick S. (2005). Slaved diffusion in phospholipid bilayers. Proc. Natl. Acad. Sci. USA.

[59] Netz R.R., Andelman D. (2003). Neutral and charged polymers at interfaces. Phys. Rep..

[60] Knight J.D., Lerner M.G., Marcano-Velazquez J.G., Pastor R.W., Falke J.J. (2010). Single molecule diffusion of membrane-bound proteins: Window into lipid contacts and bilayer dynamics. Biophys. J..

[61] Ziemba B.P., Falke J.J. (2013). Lateral diffusion of peripheral membrane proteins on supported lipid bilayers is controlled by the additive frictional drags of (1) bound lipids and (2) protein domains penetrating into the bilayer hydrocarbon core. Chem. Phys. Lipids.

[62] Camley B.A., Brown F.L.H. (2013). Diffusion of complex objects embedded in free and supported lipid bilayer membranes: Role of shape anisotropy and leaflet structure. Soft Matter.

